# Voxel-Based Meta-Analysis of Gray Matter Abnormalities in Multiple System Atrophy

**DOI:** 10.3389/fnagi.2020.591666

**Published:** 2020-11-27

**Authors:** Junyu Lin, Xinran Xu, Yanbing Hou, Jing Yang, Huifang Shang

**Affiliations:** Laboratory of Neurodegenerative Disorders, Department of Neurology, Rare Diseases Center, West China Hospital, Sichuan University, Chengdu, China

**Keywords:** multiple system atrophy, voxel-based morphometry, gray matter volume, meta-analysis, subtype

## Abstract

**Purpose:** This study aimed to identify consistent gray matter volume (GMV) changes in the two subtypes of multiple system atrophy (MSA), including parkinsonism subtype (MSA-P), and cerebellar subtype (MSA-C), by conducting a voxel-wise meta-analysis of whole brain voxel-based morphometry (VBM) studies.

**Method:** VBM studies comparing MSA-P or MSA-C and healthy controls (HCs) were systematically searched in the PubMed, Embase, and Web of Science published from 1974 to 20 October 2020. A quantitative meta-analysis of VBM studies on MSA-P or MSA-C was performed using the effect size-based signed differential mapping (ES-SDM) method separately. A complementary analysis was conducted using the Seed-based d Mapping with Permutation of Subject Images (SDM-PSI) method, which allows a familywise error rate (FWE) correction for multiple comparisons of the results, for further validation of the results.

**Results:** Ten studies were included in the meta-analysis of MSA-P subtype, comprising 136 MSA-P patients and 211 HCs. Five studies were included in the meta-analysis of MSA-C subtype, comprising 89 MSA-C patients and 134 HCs. Cerebellum atrophy was detected in both MSA-P and MSA-C, whereas basal ganglia atrophy was only detected in MSA-P. Cerebral cortex atrophy was detected in both subtypes, with predominant impairment of the superior temporal gyrus, inferior frontal gyrus, temporal pole, insula, and amygdala in MSA-P and predominant impairment of the superior temporal gyrus, middle temporal gyrus, fusiform gyrus, and lingual gyrus in MSA-C. Most of these results survived the FWE correction in the complementary analysis, except for the bilateral amygdala and the left caudate nucleus in MSA-P, and the right superior temporal gyrus and the right middle temporal gyrus in MSA-C. These findings remained robust in the jackknife sensitivity analysis, and no significant heterogeneity was detected.

**Conclusion:** A different pattern of brain atrophy between MSA-P and MSA-C detected in the current study was in line with clinical manifestations and provided the evidence of the pathophysiology of the two subtypes of MSA.

## Introduction

Multiple system atrophy (MSA) is a sporadic disorder characterized by autonomic dysfunction in combination with parkinsonism and/or cerebellar ataxia. According to the predominant motor symptom, MSA is categorized into parkinsonism subtype (MSA-P) and cerebellar subtype (MSA-C). MSA has been pathologically confirmed to be an α-synucleinopathy, as the α-synuclein-positive glial cytoplasmic inclusions (GCIs) in various areas of the central nervous system, leading to subsequent neuronal death and reactive astrogliosis (Fanciulli and Wenning, 2015). As an atypical parkinsonian syndrome, MSA is thought to have more extensive brain structure abnormalities than Parkinson's disease (PD). However, the affected areas and the exact morphological changes in the brain of the two subtypes of MSA remain unclear.

Voxel-based morphometry (VBM) is a semi-automated, time-efficient, operator independent, and unbiased analytical technique, which allows detection of regional morphological changes in the whole brain *in vivo* (Ashburner and Friston, 2000). A number of VBM studies have been conducted to explore the gray matter volume (GMV) differences between the MSA-P/MSA-C patients and the healthy controls (HCs) recently. However, these studies have yield inconsistent results. For example, some studies found cerebellum atrophy in MSA-P (Minnerop et al., 2007; Chang et al., 2009; Tzarouchi et al., 2010; Kim et al., 2015; Planetta et al., 2015), whereas some other studies did not (Brenneis et al., 2003; Tir et al., 2009; Wang et al., 2011; Shigemoto et al., 2013; Dash et al., 2019). Some studies detected supratentorial GMV reduction in MSA-C (Brenneis et al., 2006; Minnerop et al., 2007; Chang et al., 2009), while some other studies detected GMV reduction limited to the cerebellum in MSA-C (Specht et al., 2005; Dash et al., 2019).

The conflicting results may partially ascribe to a relatively small sample size of each study or heterogeneous subjects enrolled in each study. Therefore, a quantitative meta-analysis of these VBM studies is of significance to identify consistent GMV changes in each subtype of MSA. Two studies have conducted meta-analysis comparing GMV between MSA-P and HCs using anatomic likelihood estimation (ALE) method in 2015 (Shao et al., 2015; Yu et al., 2015). However, there were only five or six literature included, and no studies comparing GMV between MSA-C and HCs were analyzed. The effect size-based signed differential mapping (ES-SDM) is a newly developed meta-analytic method based on well-established statistics accounting for within- and between-study variance, which allows combining both peak coordinates and statistical parametric maps. ES-SDM has been shown to be valid and superior to ALE method with higher sensitivity (Radua et al., 2012). Therefore, the present study aims to voxel-wisely meta-analyze the GMV changes in MSA-P and MSA-C patients using ES-SDM method. Since the updated version of ES-SDM, namely, the Seed-based d Mapping with Permutation of Subject Images (SDM-PSI) allows a familywise error rate (FWE) correction for multiple comparisons of the results (Albajes-Eizagirre et al., 2019), we will conduct a complementary analysis using the SDM-PSI method for further validation of the results.

## Materials and Methods

### Searching Method, Quality Assessment, and Data Extraction

Studies published from 1974 to 20 October 2020 were searched comprehensively in the PubMed, Embase, and Web of Science by two researchers (Junyu Lin and Jing Yang) independently using the combined keywords (“multiple system atrophy” OR “MSA” OR “multisystem atrophy” OR “Shy-Drager syndrome” OR “olivopontocerebellar atrophy” OR “autonomic failure” OR “striatonigral degeneration” OR “OPCA” OR “SND”) and (“VBM” OR “voxel based morphometry”). The reference list of the included articles and relevant reviews was also searched for potential inclusion.

Studies were included if they (1) conducted VBM to detect whole-brain GMV in MSA-P or MSA-C patients and HCs; (2) included MSA patients who met clinical diagnosis criteria of “probable” or “possible” MSA; (3) used significant thresholds to compare GMV differences between MSA-P or MSA-C patients and HCs; (4) reported GMV differences in a standard stereotactic space [Talairach or Montreal Neurological Institute (MNI)] with three-dimensional coordinates (*x, y, z*) or did not find significant differences; and (5) were published in English. Studies were excluded if (1) there were no HCs; (2) the stereotactic coordinates were not available, even if we contacted the authors to ask for help; (3) the results were only reported in regions of interest (ROIs) instead of in the whole brain; (4) MSA patients were studied as one group without distinguishing MSA-P or MSA-C; and (5) the study was conducted in the same center as previously published using overlapping data. In this case, the study with the largest sample size was selected.

A 15-point checklist (Supplementary Figure 1) was used to assess the quality of the articles ready for inclusion. Developed based on previous studies (Shepherd et al., 2012; Iwabuchi et al., 2015), the checklist comprised a comprehensive evaluation of sample characteristics and imaging-specific methodology.

In each included study, peak coordinates and their effect sizes (*t*-values, *z* scores, or *p*-values) with significant differences between MSA-P/MSA-C and HCs in GM volume were extracted according to the SDM tutorial. Literature searching and data extraction were performed by two neurologists (Junyu Lin and Jing Yang) independently. If there were disagreements, the third neurologist (XX) helped to check the data and make a decision.

### Meta-Analysis of Voxel-Based Morphometry Studies

The meta-analysis was performed following the ES-SDM tutorial using SDM software package (www.sdmproject.com). The approach has been described in detail previously (Radua et al., 2012). The analyses of MSA-P and MSA-C were performed separately using the same method. First, a file containing samples sizes, coordinates, effect sizes, and clinical characteristics (e.g., mean age and disease duration of MSA patients) was created. Then, a mean analysis was conducted to compare the GMV between MSA-P/MSA-C and HCs. The default kernel size and statistical thresholds [full width at half maximum (FWHM) = 20 mm, *p* = 0.005, peak height threshold = 1, extent threshold = 10 voxels] were used, which have been validated to optimize the sensitivity and specificity and to produce a desirable balance between Type I and II error rates (Lieberman and Cunningham, 2009; Radua et al., 2012).

Q statistics were calculated to assess the heterogeneity between studies. Egger's tests were carried out to detect potential publication bias, and funnel plots were established for visual inspection. *p* < 0.05 and an asymmetric plot were recognized as significant. Jackknife sensitivity analyses were conducted to assess the robustness of the main meta-analytical output by removing one study at a time and repeating the analysis.

Finally, meta-regression analyses were carried out to examine the potential confounding variables such as age, disease duration, Unified Parkinson's Disease Rating Scale III (UPDRS-III) scores, and Mini-Mental State Examination (MMSE) scores using a stringent threshold (*p* = 0.0005, extent threshold = 10 voxels). Subgroup analyses were conducted if necessary.

Complementary analyses were conducted using the SDM-PSI software version 6.21 (www.sdmproject.com), with a threshold of FWE-corrected *p* < 0.05. *I*^2^ statistics were calculated to assess the heterogeneity between studies (*I*^2^ > 50% indicates serious heterogeneity). Egger's tests were calculated to assess potential publication bias (*p* < 0.05 indicates obvious publication bias).

## Results

### Included Studies

**Figure 1:**
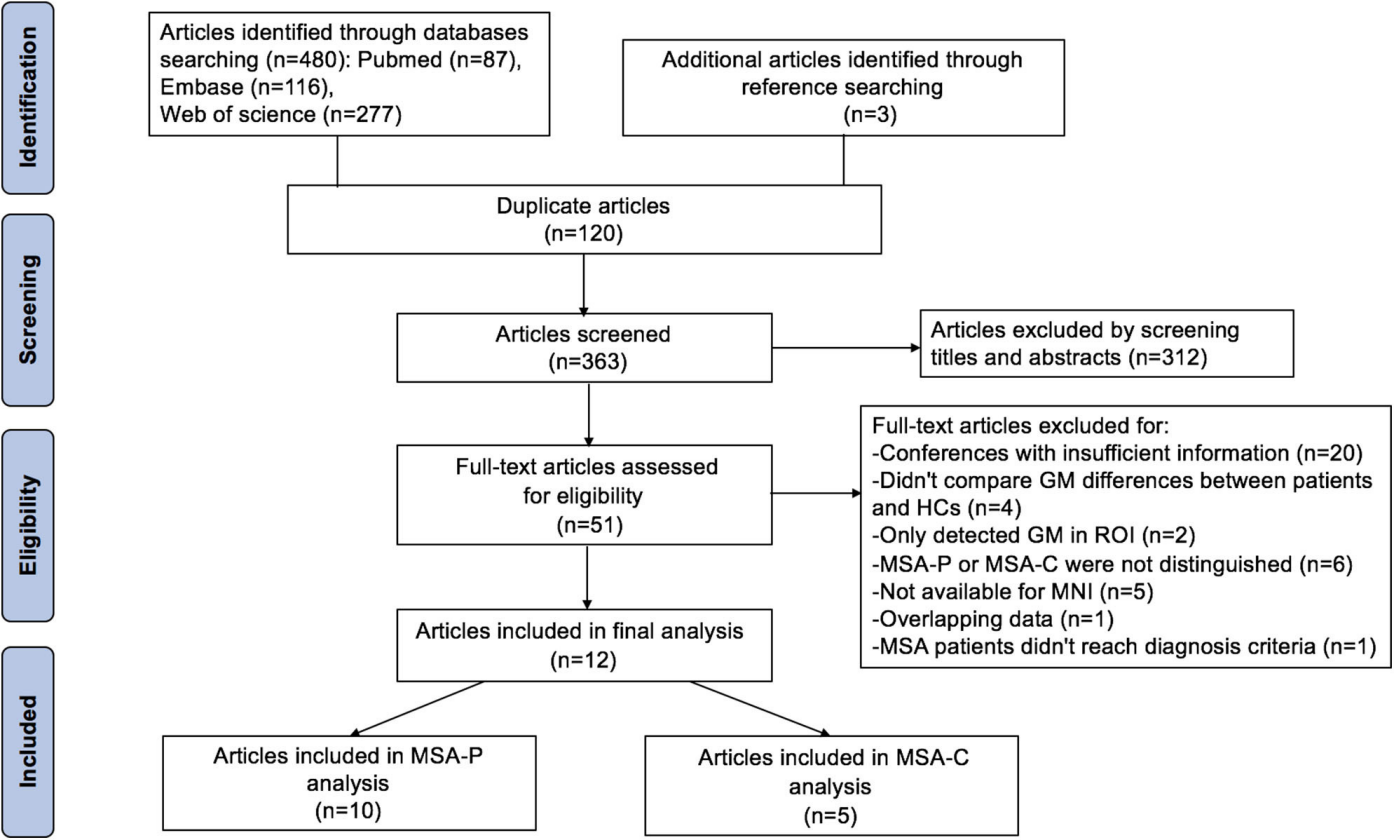
Flow chart for the search process.

The search strategy yielded 480 potentially relevant studies initially, and three additional studies were identified through references searching. Twelve studies that met the inclusion criteria were finally included in the meta-analysis. The quality scores of these studies were higher than 13 scores (Table 1). The detailed identification and attrition of studies are shown in the Figure 1. Among the 12 studies (Brenneis et al., 2003, 2006; Specht et al., 2005; Minnerop et al., 2007; Chang et al., 2009; Tir et al., 2009; Tzarouchi et al., 2010; Wang et al., 2011; Shigemoto et al., 2013; Kim et al., 2015; Planetta et al., 2015; Dash et al., 2019), 10 studies (Brenneis et al., 2003; Minnerop et al., 2007; Chang et al., 2009; Tir et al., 2009; Tzarouchi et al., 2010; Wang et al., 2011; Shigemoto et al., 2013; Kim et al., 2015; Planetta et al., 2015; Dash et al., 2019) detected the GMV differences between the MSA-P patients and the HCs, comprising 136 MSA-P patients and 211 HCs; five studies (Specht et al., 2005; Brenneis et al., 2006; Minnerop et al., 2007; Chang et al., 2009; Dash et al., 2019) detected the GMV differences between the MSA-C patients and the HCs, comprising 89 MSA-C patients and 134 HCs. The age and sex were matched between patients and HCs in each study. The demographic and clinical characteristics of the included studies are summarized in Table 1.

**Table 1:** Characteristics of included VBM studies in the current meta-analysis.

**Study**	**Sample**	**Sex (M/F)**	**Age (years)**	**Disease duration (years)**	**UPDRS-III scores**	**MMSE scores**	**Scanner (T)**	**Software**	**Threshold**	**Quality scores**
**MSA-P**										
Brenneis et al. (2003)	MSA-P 12	NA	62 , 6.6	2.8 ± 1.1	42	NA	1.5	SPM99	*p* < 0.05 corrected	13
	HC 12	NA	60 , 5.8							
Kim et al. (2015)	MSA-P 15	8/7	65.27 , 9.68	2.6 ± 1.59	30.30 ± 9.23	27.53 ± 1.64	3	FSL	*p* < 0.05 corrected	14
	HC 32	19/13	66.97 , 5.09							
Planetta et al. (2015)	MSA-P 14	8/6	64.6 , 9.0	6.5 ± 2.7	36.4 ± 13.0	27.4 ± 2.3	3	SPM8	*p* < 0.05 corrected	15
	HC 14	9/5	61.9 , 8.4							
Shigemoto et al. (2013)	MSA-P 20	7/13	62.9 , 7.7	4.1 ± 2.2	NA	NA	1.5	SPM8	*p* < 0.05 corrected	15
	HC 30	10/20	64.7 , 7.7							
Tir et al. (2009)	MSA-P 14	5/9	63.6 , 9.74	5.1 ± 2.2	39 ± 17	NA	1.5	SPM2	*p* < 0.05 corrected	14
	HC 14	5/9	61.6 , 7.6							
Tzarouchi et al. (2010)	MSA-P 11	9/2	61.9 , 11.7	5.42 ± 2.5	NA	NA	1.5	SPM	*p* < 0.001 uncorrected	13
	HC 11	8/3	64.63 , 10.4							
Wang et al. (2011)	MSA-P 12	4/8	63.0 , 12.7	5.4 ± 2.8	NA	NA	1.5	SPM2	*p* < 0.05 corrected	14
	HC 20	10/10	52.4 , 19.5							
Chang et al. (2009)	MSA-P 13	9/4	59.8 , 8.1	2.6	NA	24.9 ± 5.3	3	SPM2	*p* < 0.01 corrected	15
	HC 37	20/17	55.5 , 8.6							
Dash et al. (2019)	MSA-P 9	5/4	53.8 , 6.0	1.5 ± 1.0	NA	NA	3	SPM8	*p* < 0.05 corrected	13
	HC 25	18/7	55.0 , 6.8							
Minnerop et al. (2007)	MSA-P 16	8/8	62.8 , 5.6	4.4 ± 2.3	NA	NA	1.5	SPM2	*p* < 0.01 corrected	14
	HC 16	8/8	62.3 , 4.3							
**MSA-C**										
Chang et al. (2009)	MSA-C 10	5/5	57.1 , 9.9	2.4	NA	21 ± 5.7	3	SPM2	*p* < 0.01 corrected	15
	HC 37	20/17	55.5 , 8.6							
Dash et al. (2019)	MSA-C 20	10/10	55.7 , 5.4	1.7 ± 0.8	NA	NA	3	SPM8	*p* < 0.05 corrected	14
	HC 25	18/7	55.0 , 6.8							
Minnerop et al. (2007)	MSA-C 32	19/13	60.5 , 6.2	4.5 ± 2.4	NA	NA	1.5	SPM2	*p* < 0.01 corrected	14
	HC 46	22/24	58.7 , 6.1							
Brenneis et al. (2006)	MSA-C 13	8/5	61.3 , 6.2	3.6 ± 1.4	NA	NA	1.5	SPM99	*p* < 0.05 corrected	14
	HC 13	8/5	60.5 , 4.4							
Specht et al. (2005)	MSA-C 14	5/9	59.4 , 7.4	3.7 ± 1.4	NA	NA	1.5	SPM99	*p* < 0.05 corrected	14
	HC 13	5/8	55.1 , 6.9							

*VBM, voxel-based morphometry; UPDRS-III, Unified Parkinson's Disease Rating Scale III; MMSE, Mini-Mental State Examination; MSA, multiple system atrophy; MSA-P, multiple system atrophy with predominant parkinsonism; MSA-C, multiple system atrophy with predominant cerebellar ataxia; NA, none reported*.

### Regional Differences in Gray Matter Volume

**Figure 2:**
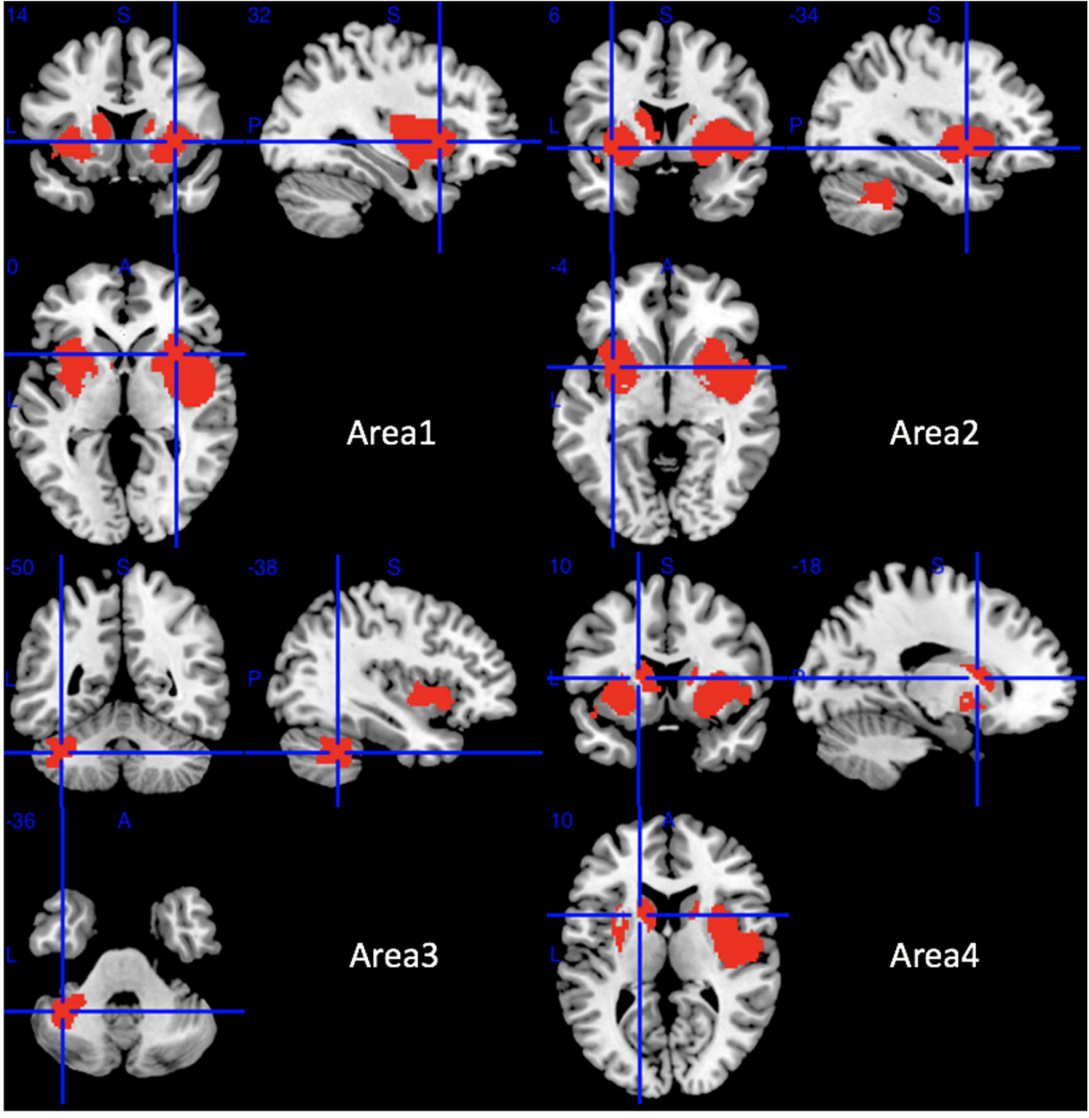
Regions of gray matter decrease in patients with MSA-P compared with healthy controls. MSA-P, multiple system atrophy with predominant parkinsonism.

Compared with HCs, MSA-P patients showed significant regional GMV reductions in the bilateral insula, the bilateral lenticular nucleus and putamen, the bilateral striatum, the right rolandic operculum (RO), the right Heschl gyrus, the right superior temporal gyrus, the opercular part of the right inferior frontal gyrus, the bilateral amygdala, the left cerebellar hemispheric lobule (VI, VIIB, and VIII), the left cerebellar crus I and II, and the left caudate nucleus (Table 2 and Figure 2).

**Table 2:** The mean meta-analysis: gray matter volume reductions in MSA-P patients relative to HCs.

**Regions**	**No. of voxels**	**Maximum MNI coordinates (*x, y, z*)**	**SDM-Z value**	***p*-value**	**Egger test (*p*-value)**	**Clusters' breakdown**	**Jackknife sensitivity analysis**
Area 1	3,210	32, 14, −6	−3.425	~0	0.965	Right insula Right lenticular nucleus and putamen Right striatum Right rolandic operculum (RO) Right Heschl gyrus Right superior temporal gyrus Right inferior frontal gyrus, opercular part Right amygdala Right temporal pole, superior temporal gyrus	10 out of 10 10 out of 10 10 out of 10 10 out of 10 8 out of 10 9 out of 10 10 out of 10 10 out of 10 9 out of 10
Area 2	1,459	−34, 6, −4	−2.908	<0.0001	0.828	Left insula, Left lenticular nucleus and putamen, Left striatum, Left amygdala	10 out of 10 10 out of 10 10 out of 10 10 out of 10
Area 3	587	−38, −50, −36	−2.612	<0.0001	0.010	Left cerebellar hemispheric lobule VI, Left cerebellar hemispheric lobule VIIB, Left cerebellar hemispheric lobule VIII, Left cerebellar crus I, Left cerebellar crus II	9 out of 10 8 out of 10 7 out of 10 9 out of 10 8 out of 10
Area 4	389	−18, 10, 10	−2.519	0.0001	0.049	Left caudate nucleus	10 out of 10

*MSA-P, multiple system atrophy with predominant parkinsonism; HCs, healthy controls; MNI, Montreal Neurological Institute; SDM, Seed-based d Mapping*.

**Figure 3:**
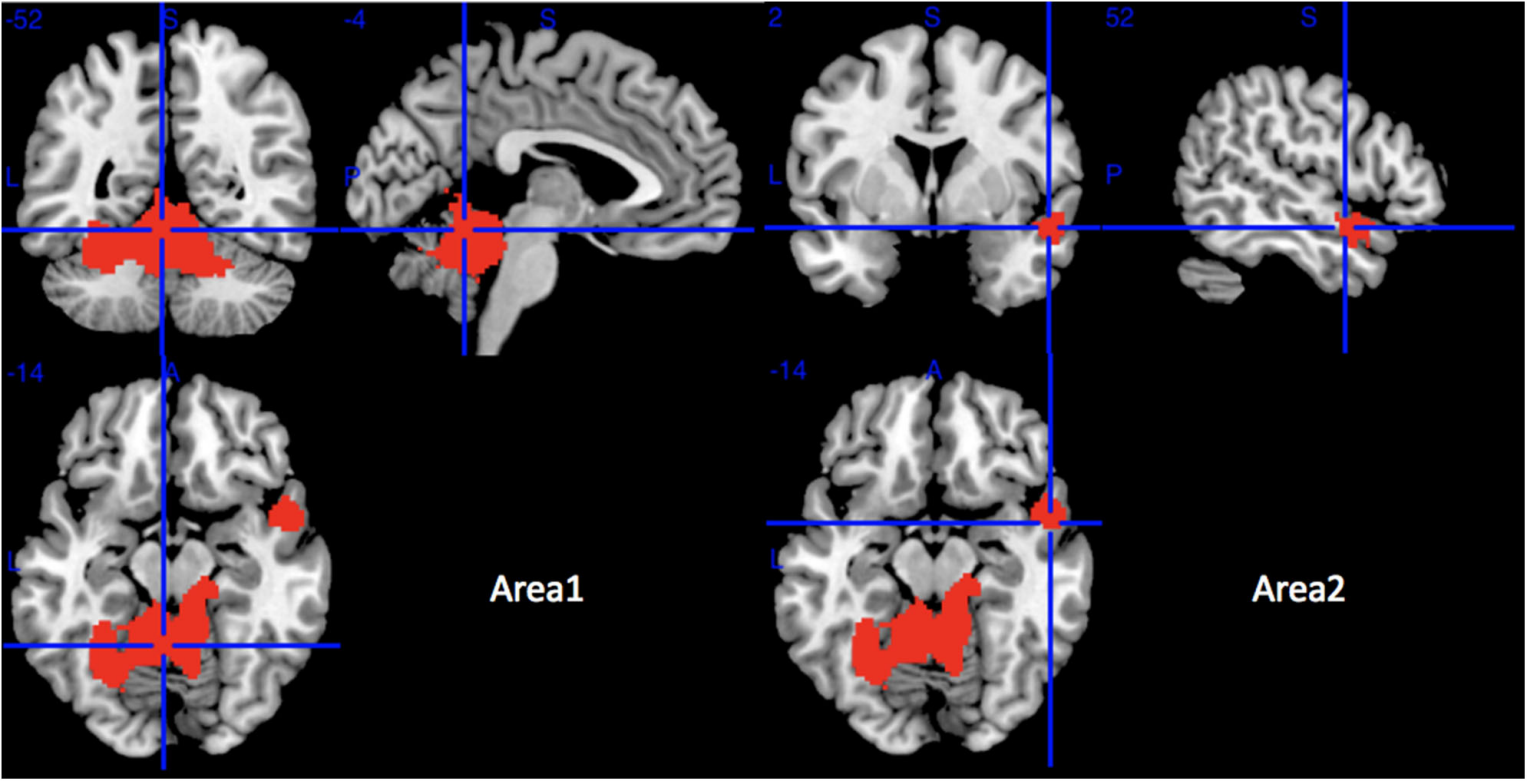
Regions of gray matter decrease in patients with MSA-C compared with healthy controls. MSA-C, multiple system atrophy with predominant cerebellar ataxia.

Compared with HCs, MSA-C patients showed significant regional GMV reductions in the bilateral cerebellar hemispheric lobule III–VI, the left cerebellar crus I, the cerebellar vermic lobule (III–VI, VIII and X), the left fusiform gyrus, the bilateral lingual gyrus, the right superior temporal gyrus, and the right middle temporal gyrus (Table 3 and Figure 3). No significant regional GMV increase was detected in MSA-P or MSA-C patients.

**Table 3:** The mean meta-analysis: gray matter volume reductions in MSA-C patients relative to HCs.

**Regions**	**No. of voxels**	**Maximum MNI coordinates (*x, y, z*)**	**SDM-Z value**	***p*-value**	**Egger test (*p*-value)**	**Clusters' breakdown**	**Jackknife sensitivity analysis**
Area 1	5,384	−4, −52, −14	−5.307	~0	0.917	Left cerebellar hemispheric lobule III Left cerebellar hemispheric lobule IV/V Left cerebellar hemispheric lobule VI Right cerebellar hemispheric lobule III Right cerebellar hemispheric lobule IV/V Right cerebellar hemispheric lobule VI Left cerebellar crus I Cerebellar vermic lobule III Cerebellar vermic lobule IV/V Cerebellar vermic lobule VI Cerebellar vermic lobule VIII Cerebellar vermic lobule X Left fusiform gyrus Left lingual gyrus Right lingual gyrus	5 out of 5 5 out of 5 5 out of 5 5 out of 5 5 out of 5 5 out of 5 5 out of 5 5 out of 5 5 out of 5 5 out of 5 5 out of 5 5 out of 5 5 out of 5 5 out of 5 5 out of 5
Area 2	269	52, 2, −14	−2.667	<0.001	0.673	Right temporal pole, superior temporal gyrus Right temporal pole, middle temporal gyrus Right superior temporal gyrus	3 out of 5 3 out of 5 3 out of 5

*MSA-C, multiple system atrophy with predominant cerebellar ataxia; HCs, healthy controls; MNI, Montreal Neurological Institute; SDM, Seed-based d Mapping*.

### Analyses of Sensitivity, Heterogeneity, and Publication Bias

The jackknife sensitivity analysis revealed that all the abovementioned regional differences were highly robust in both MSA-P and MSA-C groups (Tables 2, 3). Heterogeneity analysis using Q statistics indicated that there was no variability between studies. The funnel plots showed no obvious asymmetric of all significant brain regions. However, the quantitative assessment measured by Egger's tests revealed publication bias in two areas in MSA-P (Table 2).

### Meta-Regression Analyses

Meta-regression analyses were conducted in the MSA-P and MSA-C groups separately. In the MSA-P group, there was an increased probability of finding brain atrophy in bilateral insula and right cerebellar crus with lower MMSE score. Age, disease duration, and UPDRS-III score did not have any significant effect on the observed between-group GMV differences. In the MSA-C group, both increased age and a longer disease duration were correlated with severe GM atrophy in the cerebellar vermic lobule X.

### Complementary Analyses

In the complementary analyses, using a threshold of FWE-corrected *p* < 0.05, most of the results overlapped with the uncorrected results. However, in MSA-P, the bilateral amygdala and the left caudate nucleus did not survive the FWE correction, while the left fusiform gyrus showed significant regional GMV reductions after the FWE correction. In MSA-C, the right superior temporal gyrus and the right middle temporal gyrus did not survive the FWE correction. The *I*^2^ statistics (*I*^2^ = 2.99–12.68%) and Egger's tests (*p* = 0.333–0.930) showed low heterogeneity and no obvious publication bias. The results are shown in Supplementary Tables 1, 2 and Supplementary Figures 2, 3.

## Discussion

The current meta-analysis revealed a different pattern of GMV reduction between the two subtypes. Besides cerebellum atrophy detected in both MSA-P and MSA-C, basal ganglia atrophy was only detected in MSA-P. GMV reduction in the cerebral cortex is different between the two subtypes.

### Basal Ganglia and the Cerebellum

Although the mainly affected areas are thought to be different between the MSA-P and MSA-C subtypes (Ozawa et al., 2004), evidence arose that there are overlapping affected areas in the two subtypes. For example, basal ganglia involvement has also been reported in MSA-C subtype, while cerebellum involvement has also been reported in MSA-P subtype (Schulz et al., 1994, 1999; Wang et al., 2011). In the current meta-analysis, GMV reductions were detected in the cerebellum in both the two subtypes, whereas GMV reduction of the basal ganglia was only detected in the MSA-P not in MSA-C subtype.

In line with the current meta-analysis, basal ganglia abnormalities have been widely reported in patients with MSA-P by previous pathological (Goto et al., 1996; Wenning et al., 2002; Ozawa et al., 2004), volumetric MRI (Schulz et al., 1999; Messina et al., 2011), diffusion-weighted imaging (DWI) (Schocke et al., 2004), functional MRI (fMRI) (Planetta et al., 2015), dopamine transporter–positron emission tomography (DAT-PET) (Ghaemi et al., 2002), and fluorodeoxyglucose–positron emission tomography (FDG-PET) (De Volder et al., 1989; Juh et al., 2005; Lyoo et al., 2008; Baudrexel et al., 2014) studies. The atrophy of the basal ganglia observed in MSA-P accounts for the poor response to levodopa, which is a key characteristic for MSA-P (Fanciulli and Wenning, 2015). Inconsistent finding of basal ganglia atrophy in MSA-C has been reported by some pathological (Ozawa et al., 2004; Jellinger et al., 2005) and quantitative MRI studies (Schulz et al., 1994, 1999; Minnerop et al., 2007; Dash et al., 2019). The current meta-analysis did not find basal ganglia atrophy in MSA-C. A pathological study (Ozawa et al., 2004) found that relatively mild atrophy of the basal ganglia could cause clinically manifestation of parkinsonism, while more advanced cerebellar atrophy was required for ataxia symptom, which could partially explain the findings of our study due to a shorter disease duration of the included MSA-C patients (mean duration 3.7 years) compared with that of the included MSA-P patients (mean duration 4.1 years). The other possible explanation was the lack of sensitivity of VBM technique in detecting GMV changes in the basal ganglia due to the subcortical localization and the small volume of the basal ganglia (Minnerop et al., 2007). A recent quantitative susceptibility mapping (QSM) study detected higher QSM values in the substantia nigra in MSA-C compared with HCs (Sugiyama et al., 2019), indicating that more sensitive methods are needed to explore whether the basal ganglia would be affected in MSA-C.

Cerebellum abnormalities of MSA-C have been widely reported by previous pathological (Wenning et al., 1997; Ozawa et al., 2004), volumetric MRI (Schulz et al., 1994, 1999), diffusion tensor imaging (DTI) (Wang et al., 2011), PET (Juh et al., 2005), and QSM (Sugiyama et al., 2019) studies. In the current meta-analysis, GMV reductions were found in the cerebellar hemispheric lobule III–VI, cerebellar crus I, and cerebellar vermic lobule (III–VI, VIII, and X) in MSA-C. Atrophy of cerebellar hemispheric lobule III–V may result in ataxic symptoms, which are prominent symptoms in MSA-C since these regions have been reported to dominate sensorimotor control (Stoodley and Schmahmann, 2010; Stoodley et al., 2012). Atrophy of cerebellar hemispheric lobule VI and crus I observed in our study may be partially in charge of the cognitive impairment detected in MSA-C patients (Santangelo et al., 2020a) since these areas have been reported to be associated with language, verbal working memory, spatial processing, executive functions, and emotional control (Stoodley and Schmahmann, 2009, 2010; Stoodley et al., 2012). A previous VBM study also revealed that anterior cerebellar (cerebellar hemispheric lobule IV–V) volume was negatively correlated with motor performance, whereas posterior lobe integrity was positively correlated with cognitive assessment in MSA-C (Yang et al., 2019). Cerebellar vermic lobule IV–IX in combination with cerebellar hemispheric lobule VI/VII have been reported to participate in control of eye movements (Jenkinson and Miall, 2010). Atrophy of these regions may be responsible for abnormal eye movements in MSA-C patients (Anderson et al., 2008). In addition, the current meta-regression analysis revealed a positive correlation between GM atrophy of cerebellar and disease duration, which was in accordance with a previous VBM-correlation analysis (Minnerop et al., 2007) and pathological study (Wenning et al., 1997). Thus, cerebellar size may be a structural marker of disease duration in MSA-C. Cerebellum involvement in MSA-P has been reported by neuropathological (Ozawa et al., 2004), volumetric MRI (Schulz et al., 1994, 1999; Messina et al., 2011), DTI (Wang et al., 2011), and fMRI studies (Planetta et al., 2015), which was consistent with the current finding.

### Cerebral Cortex

It remains controversial whether cortical atrophy is involved in MSA. Some pathological studies have reported the pathological change of cerebral cortex such as primary sensorimotor cortex and higher motor cortices in MSA (Papp and Lantos, 1994; Konagaya et al., 1999, 2002). However, a review of 203 pathologically proven MSA patients reported that 78.4% of the patients did not have cortical atrophy, 12.7% of the patients had mild atrophy in the cerebral cortex, and only 8.9% had moderate to severe atrophy in the cerebral cortex (Wenning et al., 1997). In the current meta-analysis, GMV reductions of the cerebral cortex were detected in both the MSA-P and MSA-C subtypes.

An important finding of the current meta-analysis was the involvement of the cortex that was associated with cognition performance. Temporal cortex, prefrontal cortex, and fusiform gyrus atrophy has been detected in MSA-P, while temporal cortex, fusiform gyrus, and lingual gyrus atrophy has been detected in MSA-C in the current meta-analysis. After FWE correction, only fusiform gyrus and lingual gyrus had significant GMV loss in MSA-C.

The detected atrophy of the temporal cortex may account for the impairment of the verbal recall and working memory observed in patients with MSA (Robbins et al., 1992). A recent study found that patients with MSA-P showed decreased cortical thickness of fronto-temporal-parietal regions and that cortical thinning in temporal correlated with global cognitive status and memory impairment (Caso et al., 2020). Another study detected decreased cerebral blood flow and functional connectivity in the temporal gyrus in MSA-C using combined arterial spin labeling (ALS) perfusion and resting-state fMRI method (Zheng et al., 2019). The GMV loss detected in the prefrontal cortex of MSA-P could explain the frontal executive dysfunction impairment in MSA patients (Robbins et al., 1992, 1994; Pillon et al., 1995; Siri et al., 2013; Zhang et al., 2019). Both neuronal loss and GCIs have been detected in the frontal gyrus of patients with MSA in previous studies (Konagaya et al., 1999, 2002). An fMRI study also detected decreased regional homogeneity (ReHo) in the lateral prefrontal cortex (You et al., 2011), and previous FDG-PET studies have detected frontal and parietotemporal glucose hypometabolism in MSA patients (De Volder et al., 1989; Juh et al., 2005; Lyoo et al., 2008). In addition, the cognitive impairment of MSA-P was significantly correlated with a decrease in prefrontal perfusion detected by single-photon emission CT (SPECT) (Kawai et al., 2008). Furthermore, a previous VBM-correlation analysis revealed that the superior and inferior frontal atrophy was significantly correlated with memory scores in MSA (Chang et al., 2009), which also supported the finding of the current study. The fusiform gyrus and lingual gyrus have been reported to encode visuospatial information (Muthukrishnan et al., 2020). The fusiform gyrus atrophy detected in both MSA-P and MSA-C subtypes was consistent with the clinical findings that visuospatial and constructional function was impaired in both MSA-P and MSA-C (Kawai et al., 2008). The fusiform gyrus and lingual gyrus atrophy detected in MSA-C in the current meta-analysis was in line with previous functional MRI and ALS studies (Ren et al., 2018; Zheng et al., 2019).

The affected brain cognition areas identified in the current meta-analysis were in line with a previous clinical study, which found that patients with MSA-P and MSA-C subtype had a different pattern of cognitive impairment: MSA-P showed severe impairment of visuospatial and constructional function, verbal fluency, and executive function, whereas MSA-C showed only visuospatial and constructional function involvement and a milder degree of impairment (Kawai et al., 2008).

### Limbic System

Atrophy of the insula and amygdala in MSA-P subtype was identified in the current meta-analysis. The insula is composed of multiple functionally distinct areas such as primary sensorimotor and somatosensory cortex representing head and neck, visceral motor cortex and sensory cortex, and language areas (Augustine, 1996). By using resting-state functional connectivity (rsFC) analysis, insula has been found to have rich connections with extensive brain structures, indicating a vital role of insula for integrating cognitive-affective, sensorimotor, and autonomic information (Cauda et al., 2011). The trajectories of lateral cholinergic projection were from the nucleus basalis of Meynert to the insula, frontoparietal operculum, and superior temporal gyrus, which encodes memory (Selden et al., 1998). The positive correlation between MMSE scores and insula GMV identified in the current meta-regression analysis was in accordance with a previous functional MRI study, which has revealed decreased FC of insula in MSA patients with cognitive impairment (Yang et al., 2020). In addition, insula in combination with the prefrontal cortex and temporal pole, whose volume was decreased in the current study, constitute a complex neuroanatomical network, namely, the theory of mind (ToM) network (Bodden et al., 2010). ToM is defined as the ability to infer other people's mental states and is therefore important in human social interaction (Bodden et al., 2010). A clinical study detected deficit of both cognitive and affective components of ToM in patients with MSA (Santangelo et al., 2020b), which confirmed the ToM dysfunction in MSA.

The amygdala is also an important structure that plays a role in cognitive-affective network (Janak and Tyke, 2015). Pathological study has shown α-synuclein and transactive response DNA-binding protein of 43 kDa (TDP-43) pathology in the amygdala of MSA (Brettschneider et al., 2018; Koga et al., 2018). Functional MRI has detected lower amplitude of low-frequency fluctuations (ALFF) in the amygdala of MSA patients (Wang et al., 2017). Abnormal FC of amygdala has been reported to be associated with depressive symptoms in patients with MSA (Zhao et al., 2018), and greater neuronal cytoplasmic inclusion (NCI) burden in the amygdala has been reported to be associated with cognitive impairment in MSA (Maeda et al., 2018). More severe cognitive, affective, and autonomic symptoms observed in MSA-P than MSA-C subtype (Kawai et al., 2008; Zhang et al., 2017) may be attributed to the presence of insula and amygdala atrophy detected in MSA-P and absence of insula and amygdala atrophy in MSA-C. However, since the atrophy of the amygdala in MSA-P did not survive FWE correction, the results should be interpreted with caution, and more studies are needed to confirm the results.

A previous study also indicated that longer disease duration is associated with more progressive and diffuse atrophy of the cerebral cortex (Tzarouchi et al., 2010). The less extensive impairment of the cerebral cortex in MSA-C may partially ascribe to a relatively short disease duration of the included patients compared with the included MSA-P patients. Another explanation may be the intrinsic character of different cerebral involvement patterns of the two subtypes. MSA has recently been hypothesized to be an oligodendroglial synucleinopathy with “prion-like” propagation of misfolded α-synuclein from neurons to oligodendroglial and cell to cell (Reyes et al., 2014; Tarutani and Hasegawa, 2019), which may give clues to the spread pattern of affected areas in MSA. For example, given that the caudate receives afferent fibers mainly from the prefrontal cortex, and ventral striatum receives afferent fibers from the orbitofrontal cortex, the ventromedial frontal pole, the uncus of the temporal lobe, and the temporal pole (Lehericy et al., 2004), we speculated that the prominent involvement of frontal cortex in MSA-P subtype may be ascribed to the connection between the basal ganglia and frontal cortex. Therefore, the affected areas of the two subtypes of MSA may spread along the connected fibers, leading to different patterns of brain involvement and symptom manifestation.

## Limitations

The current study has several limitations. First, only studies published in English language were included, and studies from which we could not extract stereotactic coordinates were excluded, which might lead to bias. Second, the heterogeneity of the methodologies in the included studies could not be completely ruled out, such as different preprocessing protocols, smoothing kernels, and statistical thresholds. For instance, we included a study using SPM99, a software where the Jacobian matrix is calculated using linear analyses, which increase the presence of false negative in the comparisons between MSA and HCs. Moreover, for studies adopting software from SPM8 backward, the inclusion of the GM threshold-based masking during the pre-processing was not mandatory. Without using this mask, the possibility to capture WM alterations and consider them as part of GM changes is high. However, we have conducted the jackknife sensitivity analyses by removing one study at a time and repeating the analysis to assess the robustness of the main meta-analytical results, which could partially compensate this limitation. Third, the diagnosis of the included patients with MSA was not confirmed by autopsy. In addition, due to the short disease duration range of patients included in this meta-analysis, the risk of misinterpreting probable WM alterations as cortical GM changes may be high.

## Conclusions

The current study revealed a different pattern of GMV reduction between the two subtypes. Besides cerebellum atrophy in both MSA-P and MSA-C, basal ganglia atrophy was only detected in MSA-P. Cerebral cortex atrophy was detected in both subtypes, with predominant involvement of the temporal cortex, prefrontal cortex, fusiform gyrus, and insula in MSA-P and predominant involvement of the fusiform gyrus and lingual gyrus in MSA-C. These findings provided morphological evidence for the pathophysiology of the two subtypes of MSA.

## Data Availability Statement

The original contributions presented in the study are included in the article/Supplementary Materials, further inquiries can be directed to the corresponding author/s.

## Author Contributions

JL contributed to conception, organization and execution, data collection and statistical analysis, and drafting of the manuscript. XX, YH, and JY contributed to the execution and data collection. HS contributed to the conception and organization, manuscript review, and critique and is responsible for the overall content as the guarantor. All authors contributed to the article and approved the submitted version.

## Conflict of Interest

The authors declare that the research was conducted in the absence of any commercial or financial relationships that could be construed as a potential conflict of interest.
