# Supplementary material for: Voxel-Based Meta-Analysis of Gray Matter Abnormalities in Multiple System Atrophy
Source: Front Aging Neurosci. 2020 Nov 27;12:591666. doi: 10.3389/fnagi.2020.591666 (PMC7729009; doi:10.3389/fnagi.2020.591666)

Supplementary Table 1 Complementary analyses: Gray matter volume reductions in MSA-P patients relative to HCs with a threshold of FWE-corrected p < 0.05

| Regions | No. of voxels | Maximum MNI coordinates (x,y,z) | SDM-Z value | P value | Egger test  (p value) | Clusters’ breakdown |
| --- | --- | --- | --- | --- | --- | --- |
| Area 1 | 2023 | 28,6,0 | -6.332 | <0.001 | 0.398 | Right insula,  Right lenticular nucleus and putamen,  Right striatum,  Right rolandic oprtculum (RO),  Right heschl gyrus,  Right superior temporal gyrus,  Right inferior frontal gyrus, opercular part,  Right temporal pole, superior temporal gyrus 7 |
| Area 2 | 903 | -24,4,-2 | -6.076 | <0.001 | 0.333 | Left insula,  Left lenticular nucleus and putamen,  Left striatum, |
| Area 3 | 411 | -4,-40,-6 | -4.466 | 0.003 | 0.800 | Left cerebellar hemispheric lobule IV/V,  Cerebellum vermic lobule IIILeft cerebellar crus I, |
| Area 4 | 64 | -42,-48,-24 | -4.180 | 0.02 | 0.930 | Left fusiform gyrus,  Left inferior temporal gyrus |

MSA-P, multiple system atrophy with predominately parkinsonism; HCs, healthy controls; MNI, Montreal Neurological Institute; SDM, Seed-based d Mapping.

Supplementary Table 2 Complementary analyses: Gray matter volume reductions in MSA-C patients relative to HCs with a threshold of FWE-corrected p < 0.05

| Regions | No. of voxels | Maximum MNI coordinates (x,y,z) | SDM-Z value | P value | Egger test  (p value) | Clusters’ breakdown |
| --- | --- | --- | --- | --- | --- | --- |
| Area 1 | 1831 | -8,-56,-18 | -5.037 | ~0 | 0.758 | Left cerebellar hemispheric lobule III,  Left cerebellar hemispheric lobule IV/V,  Left cerebellar hemispheric lobule VI,  Right cerebellar hemispheric lobule III,  Right cerebellar hemispheric lobule IV/V,  Left cerebellar crus I,  Cerebellar vermic lobule IV/V,  Cerebellar vermic lobule VI,  Left fusiform gyrus,  Left lingual gyrus |

MSA-C, multiple system atrophy with predominately cerebellar ataxia; HCs, healthy controls; MNI, Montreal Neurological Institute; SDM, Seed-based d Mapping.

Supplementary Figure 1 Criteria for quality assessment of individual studies


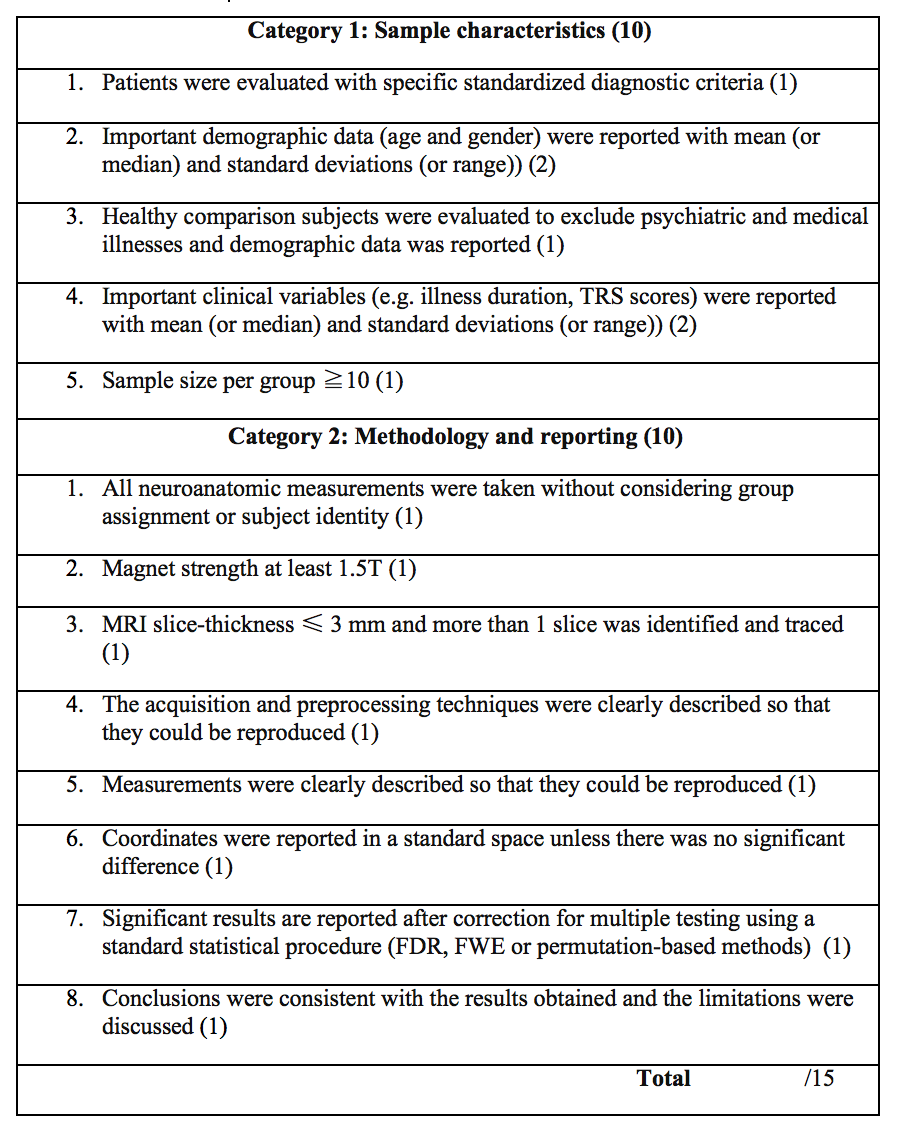


Supplementary Figure 2 Regions of gray matter decrease in patients with MSA-P compared with healthy controls with a threshold of FWE-corrected p < 0.05


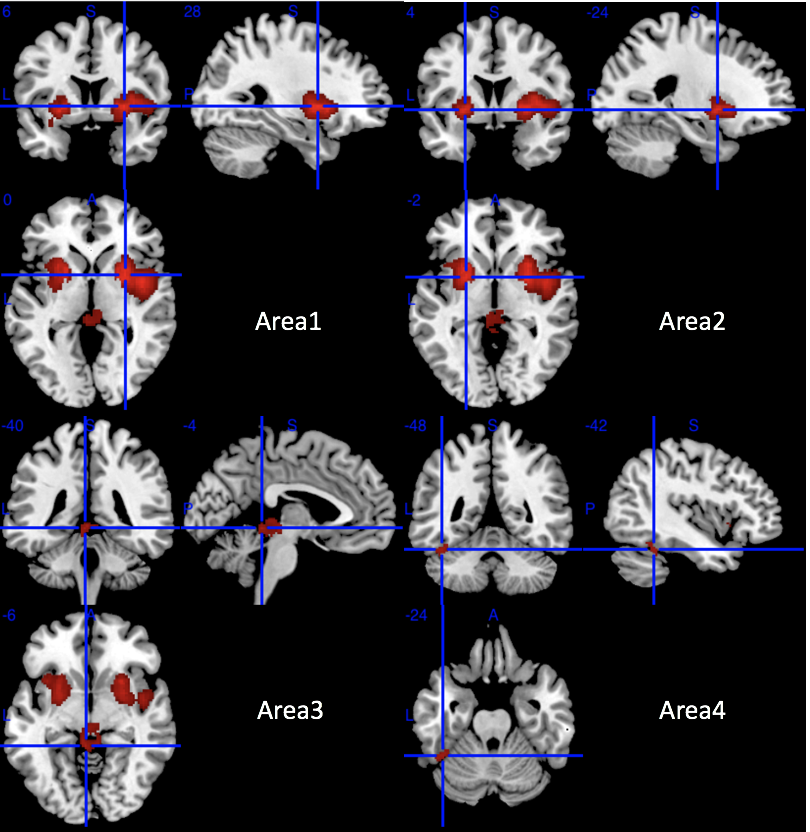


Supplementary Figure 3 Regions of gray matter decrease in patients with MSA-C compared with healthy controls with a threshold of FWE-corrected p < 0.05


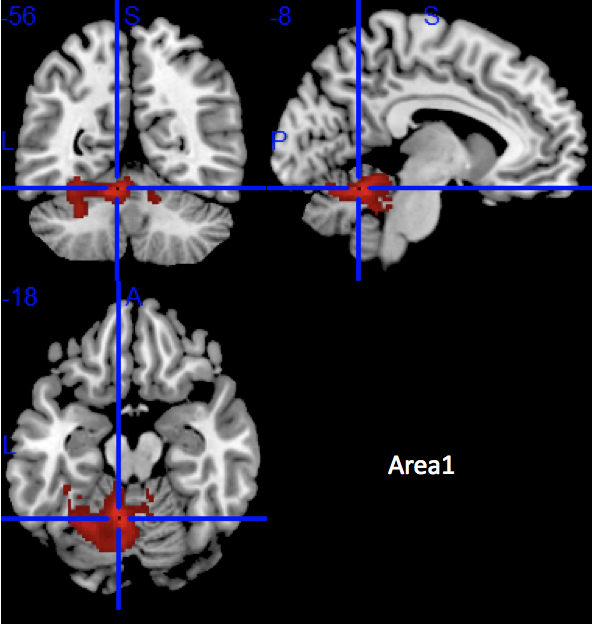

Supplement: Supplementary file 1 [file Table_1.docx]
